# Supplementary material for: Behavioral Interactions between Bacterivorous Nematodes and Predatory Bacteria in a Synthetic Community
Source: Microorganisms. 2021 Jun 23;9(7):1362. doi: 10.3390/microorganisms9071362 (PMC8307948; doi:10.3390/microorganisms9071362)
Supplement: Supplementary file 1 [file microorganisms-09-01362-s001.zip › microorganisms-1161945-supplementary.pdf]

Behavioral interactions between bacterivorous nematodes and predatory bacteria in a synthetic community

Nicola Mayrhofer [1], Gregory J. Velicer [1], Kaitlin A. Schaal [1+*] and Marie Vasse [1,2+*]

[1] Institute of Integrative Biology, ETH Zürich, 8092 Zürich, Switzerland; nicola.mayrhofer@usys.ethz.ch (N.M); gregory.velicer@env.ethz.ch (G.J.V.); kaitlin.schaal@env.ethz.ch (K.A.S.); contact@marievasse.eu (M.V.)
[2] MIVEGEC (UMR 5290 CNRS, IRD, UM), CNRS, Montpellier, France.
+ Shared last authorship. These authors contributed equally to this work.
* Correspondence: kaitlin.schaal@env.ethz.ch (K.A.S.); contact@marievasse.eu (M.V.)

Supplementary Information

Figure S1. Localization of *C. elegans* on plates containing only *M. xanthus*

Figure S2. *C. elegans* prefers prey patches not containing *M. xanthus*

Figure S3. Choice indices for prey-patch controls

Figure S4. Localization of *C. elegans* on binary choice assay plates

Table S1. Presence of prey bacteria in mixed patches

Figure S1. Localization of *C. elegans* on plates containing only *M. xanthus*. Numbers of worms in each specified plate location for the plates summarized in Figure 1 are shown. Worms that left the plates are not shown. Panel A shows the results of the binary choice assay with *M. xanthus* alone (Figs. 1A and 1B), and panel B shows the results of the half-plate choice assay (Figs. 1C and 1D). Large dots are the means of 3 biological replicates (shown as transparent dots) and error bars are standard deviations.

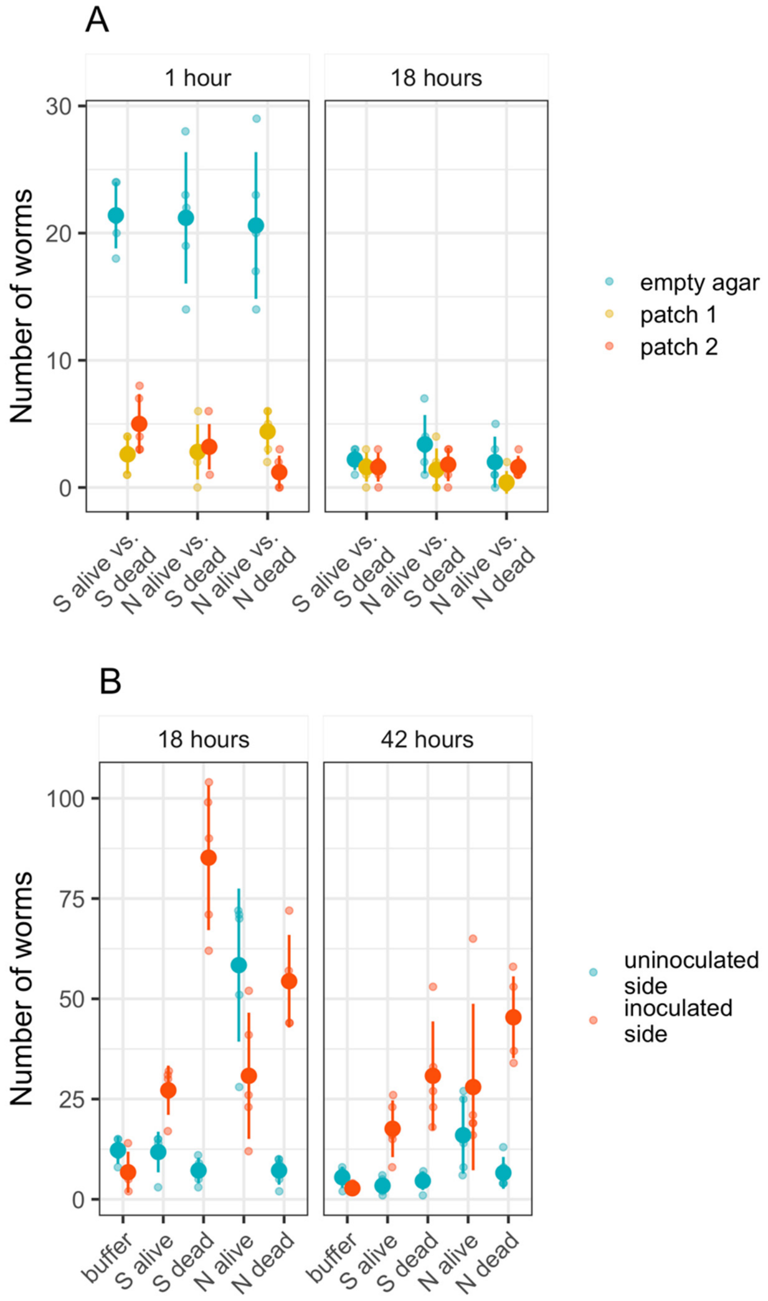

Figure S2. *C. elegans* prefers prey patches not containing *M. xanthus*. Here we show worm locations relative to two circular prey patches after 25 hours, on plates where both patches contained the same basal prey bacterium (A) or where the two patches contained different basal prey (B). Worms found on the plate but not in a patch are shown as yellow dots. Each separate panel shows the number of worms found in a single-species patch (or a control patch of buffer). Patch 1: buffer on the control plates (salmon dots), *E. coli* (dark blue dots), *F. johnsoniae* (dark red dots), or *M. xanthus* (green dots, left = strain S or right = strain N). In panel A, Patch 2 (salmon, light blue, or pink dots) contains the same basal prey bacteria as patch one, in some cases mixed together with *M. xanthus* strain S or N, as indicated on the *x*-axis. In panel B, Patch 2 contains the opposite basal prey bacterium either in pure culture (first section) or mixed with *M. xanthus* strain S or N (second and third sections). Large dots are means of 3 biological replicates (shown as transparent dots). Error bars are standard deviations. '*F. john*' = *F. johnsoniae*, '*M.xan*$_N$', '*M.xans*'= *M. xanthus* strains N and S, respectively.

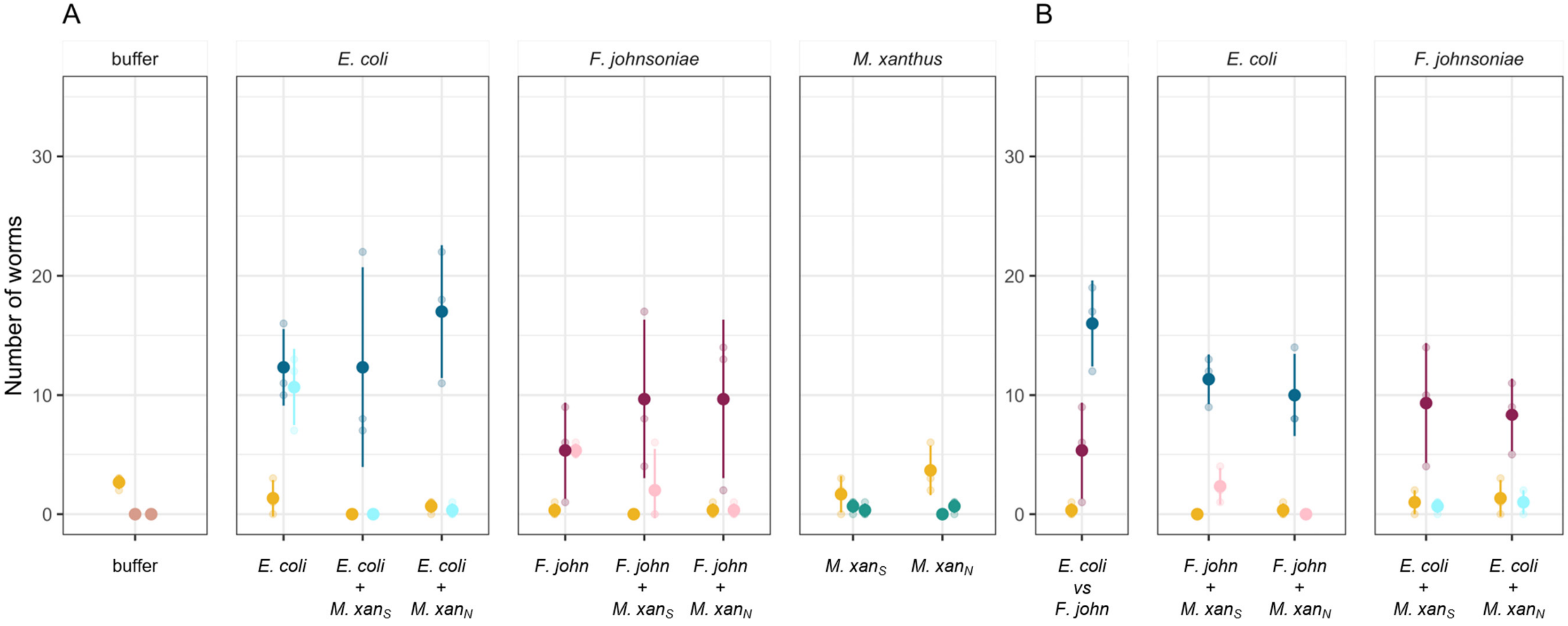

Figure S3. Choice indices for prey-patch controls. Here we show worm choice after 25 hours on control plates with two patches of the same prey. Colored bars show the means of 3 biological replicates and error bars are 95% confidence intervals. For the buffer treatment, the mean is zero and the error bars are zero. In one instance (*F. john*), 95% confidence intervals extend outside the range of what is biologically possible, [-1,1], so we restricted them to reflect the biological reality. *F. john* = *F. johnsoniae, M. xan*N / *M. xan*S = *M. xanthus* strains N and S, respectively.

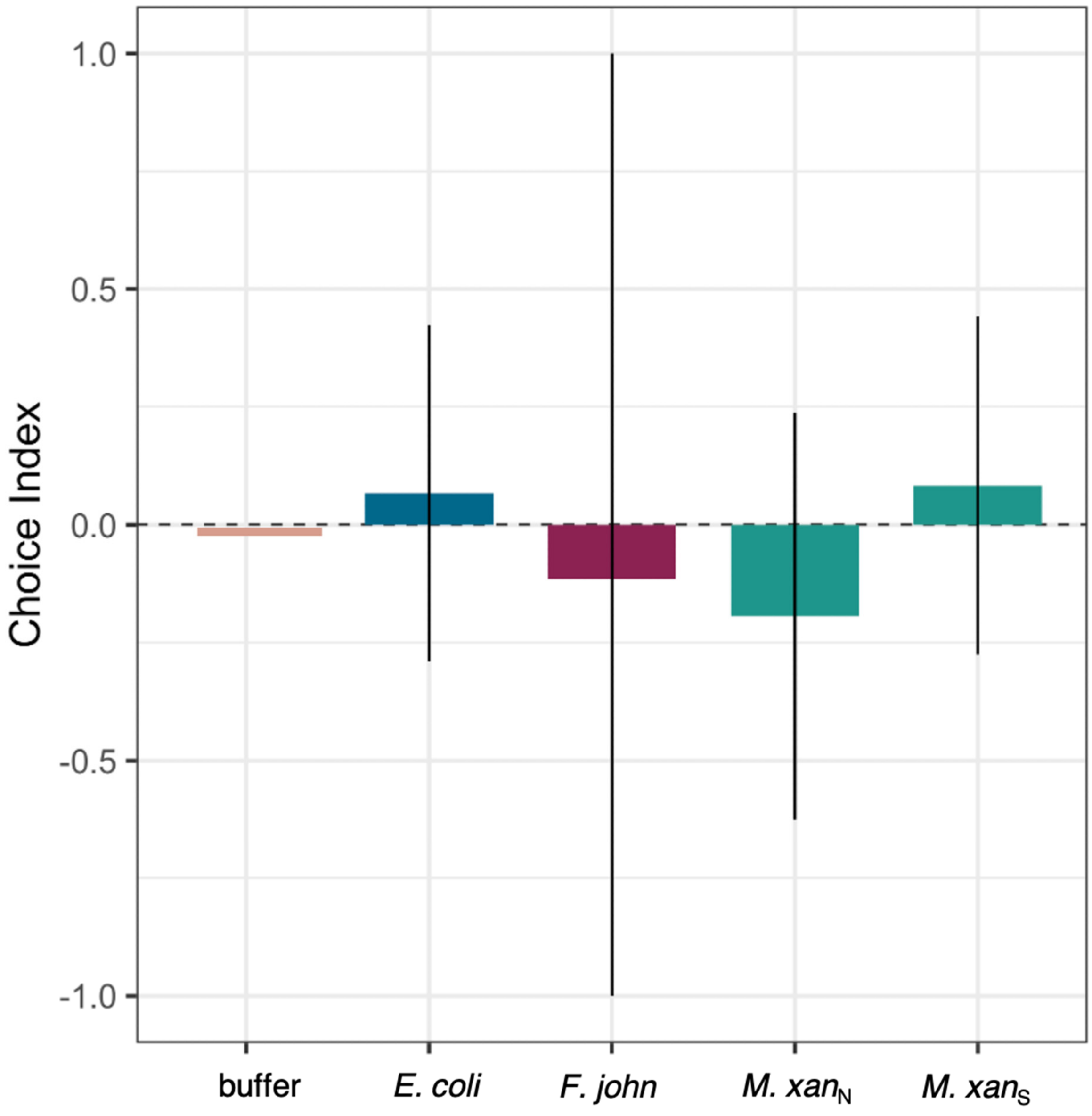

Figure S4. Localization of *C. elegans* on binary choice assay plates. Total number of worms in each location (either a prey patch, yellow/orange, or the open agar, turquoise) at three timepoints. Large dots are the means of 3 biological replicates (replicates shown as transparent dots) and error bars are standard deviations. E = *E. coli*, F = *F. johnsoniae*, Ms / MN = *M. xanthus* strains S and N, respectively. For each treatment category shown on the x axis, 'patch 1' and 'patch 2' are the first- and second-listed patch-identity indicators.

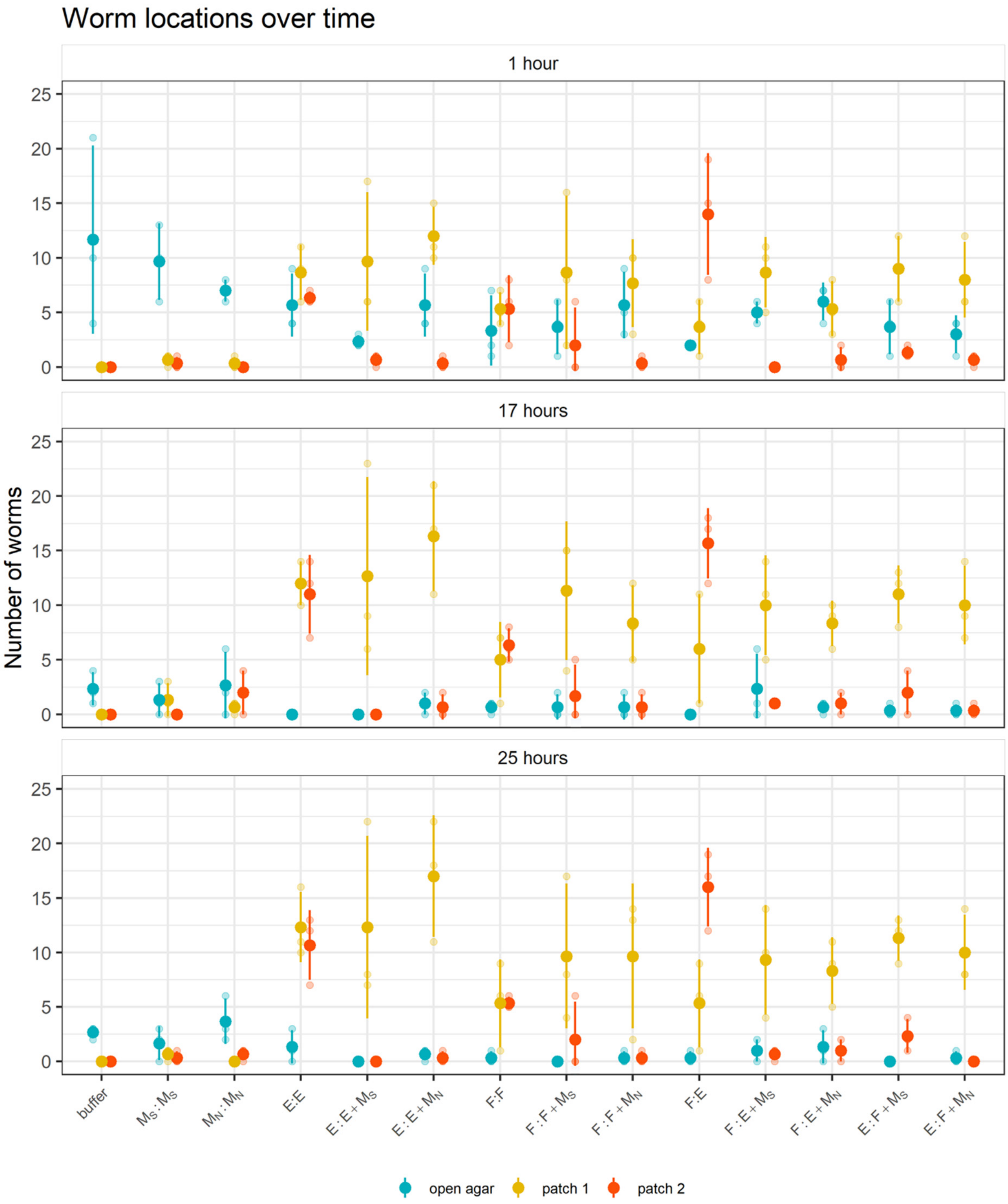

Table S1. Presence of prey bacteria in mixed patches. We tested the patches containing basal prey bacteria in the assays reported in Figure 2 to check whether basal prey bacteria could still be detected after 25 hours. The bacteria detected in each patch are shown here. Results from mixed patches where prey bacteria were still detected are indicated in bold italics. E = *E. coli*, F = *F. johnsoniae*, S = *M. xanthus* strain S, N = *M. xanthus* strain N.

Patch 1 / Patch 2	Patch 1			Patch 2		
	Replicate 1	Replicate 2	Replicate 3	Replicate 1	Replicate 2	Replicate 3
E / E	E	E	E	E	E	E
F / F	F	F	F	F	F	F
S / S	S	S	S	S	S	S
N / N	N	N	N	N	N	N
F / E	F	F	F	E	E	E
E / E+S	E	E	E	S only	S only	S only
E / E+N	E	E	E	N only	***E+N***	N
F / F+S	F	F	F	N only	S only	***F+S***
F / F+N	F	F	F	N only	N only	***F+N***
E / F+S	E	E	E	S only	***F+S***	***F+S***
E / F+N	E	E	E	N only	N only	N only
F / E+S	F	F	F	S only	S only	S only
F / E+N	F	F	F	***E+N***	***E+N***	N only
